# Cesarean delivery on maternal request: Can the ethical problem be solved by the principlist approach?

**DOI:** 10.1186/1472-6939-9-11

**Published:** 2008-06-17

**Authors:** Tore Nilstun, Marwan Habiba, Göran Lingman, Rodolfo Saracci, Monica Da Frè, Marina Cuttini, the EUROBS study group

**Affiliations:** 1 Department of Medical Ethics, University of Lund, BMC C13, SE-221 84 Lund, Sweden; 2 Reproductive Science Section, Department of Cancer Studies and Molecular Medicine, University of Leicester, Robert Kilpatrick Building, Leicester Royal Infirmary – PO Box 65, Leicester LE2 7LX, UK; 3 Department of Obstetrics and Gynaecology, Lund University, SE-223 85 Lund, Sweden; 4 IFC-National Research Council, via Trieste 41, 56100 Pisa, Italy; 5 Unit of Epidemiology, Regional Health Agency of Tuscany, Viale Milton 7, IT-50129, Florence, Italy; 6 Unit of Epidemiology, Ospedale Pediatrico Bambino Gesù, Piazza S. Onofrio 4, IT-00165 Rome, Italy

## Abstract

In this article, we use the principlist approach to identify, analyse and attempt to solve the ethical problem raised by a pregnant woman's request for cesarean delivery in absence of medical indications.

We use two different types of premises: factual (facts about cesarean delivery and specifically attitudes of obstetricians as derived from the EUROBS European study) and value premises (principles of beneficence and non-maleficence, respect for autonomy and justice).

*Beneficence/non-maleficence *entails physicians' responsibility to minimise harms and maximise benefits. Avoiding its inherent risks makes a *prima facie *case against cesarean section without medical indication. However, as vaginal delivery can have unintended consequences, there is a need to balance the somewhat dissimilar risks and benefits. The principle of *autonomy *poses a challenge in case of disagreement between the pregnant woman and the physician. Improved communication aimed to enable better informed choice may overcome some instances of disagreement. The principle of *justice *prohibits unfair discrimination, and broadly favours optimising resource utilisation.

Available evidence supports vaginal birth in uncomplicated term pregnancies as the standard of care. The principlist approach offered a useful framework for ethical analysis of cesarean delivery on maternal request, identified the rights and duties of those involved, and helped reach a conclusion, although conflict at the individual level may remain challenging.

## Background

Cesarean delivery on maternal request (CDMR), patient choice cesarean, or cesarean on demand all refer to elective caesarean section (CS) for singleton term pregnancy carried out on maternal request in the absence of maternal or fetal indications [1]. The notion can be traced back to 1985, when a provocative paper published on the *New England Journal of Medicine *suggested "prophylactic cesarean" at term to avoid the risks linked to "passive anticipation of vaginal delivery" [2]. Over a decade later, a surge of interest in the topic was prompted by the results of a survey showing that 31% of female obstetricians in London would choose a cesarean section for themselves in case of uncomplicated pregnancy [3]. Since then, CDMR has been the subject of innumerable research papers, editorials, letters, opinion surveys, reviews, guidelines and conferences [1,4-14].

Reported rates of CDMR range from 2.6% of all caesarean sections in Flanders [15] to 26.8% in Western Australia [16], and there are some indications that the rate might be increasing [17]. However, accurate figures are lacking since CDMR is neither a well-defined clinical entity, nor is coded as such in official statistics [18]. According to recent reviews [1,10,12], conclusive evidence on the risks and benefits of CDMR compared to vaginal birth is equally lacking. A case has been made for a standardized definition of CDMR and for research to validly compare CDMR with planned, rather than actual, vaginal birth [12,13,18].

This paper presents an ethical analysis of the problem posed by CDMR, carried out using the principlist approach. We assess whether the approach contributes to resolving the conflict. The analysis uses both value premises (the principles) and the findings of a European multi-centre study (EUROBS) which compared the attitudes of a large, representative sample of obstetricians in eight European countries: France, Germany, Italy, Luxembourg, the Netherlands, Spain, Sweden, and the UK [19].

### Factual and value premises

We used two different types of premises. The first are factual premises, i.e. the European obstetricians' stated attitudes to CDMR. Previous studies have shown that in ethical matters physicians' practices (i.e. "what physicians do") [20] reflect fairly closely what physicians "say they would do" [21]. Thus, for the purpose of this analysis we will assume that the reported attitudes are a valid indicator of actual behaviour.

The second are value premises, which in this case are the ethical principles formulated by Beauchamp and Childress [22]: respect for autonomy, non-maleficence, beneficence and justice. Together the value and factual premises are utilized to derive conclusions.

### Factual premises

The findings from the EUROBS study concerning doctors' attitudes to CDMR were reported elsewhere [19]. Obstetricians with at least six months experience in obstetrics were asked to complete an anonymous, self-administered questionnaire. One hundred and five obstetric units and 1530 physicians participated in the study (response rates 70% and 77% respectively).

The questionnaire included the following clinical case description: "A 25-year old pregnant woman starts labour at 39 completed weeks. The foetus is apparently normally formed, healthy, and in cephalic presentation. Despite being informed that a vaginal delivery is indicated, and of the higher morbidity and mortality associated with cesarean delivery, the woman insists on cesarean section". Physicians were asked to report what they would do in response to a range of scenarios, including the case where no additional factors related to previous pregnancies or psychosocial factors were present, and where the patient's request was based simply on her personal choice. Respondents who indicated compliance with maternal request were then asked to clarify their rationale.

Results are presented in Table 1. Compliance with this woman's request for CS simply because this "was her choice" was lowest in Spain (15% of responding obstetricians), France (19%) and The Netherlands (22%) and highest in the UK (79%) and Germany (75%). Respect for patient's autonomy was the most frequently reported justification. Concerns about legal consequences linked to complications of vaginal delivery were also mentioned in all countries, although less often in the Netherlands (30%) and Sweden (31%).

**Table 1:** Respondents' attitudes towards a request for Caesarean delivery for an uncomplicated term pregnancy (weighted proportions).

	**Italy (no. 383)**		**Spain (no. 328)**		**France (no. 100)**		**Germany (no. 139)**		**Netherlands (no. 126)**		**Luxembourg (no. 15)**		**UK (no. 163)**		**Sweden (no. 383)**	
	%	(95% CI)	%	(95% CI)	%	(95% CI)	%	(95% CI)	%	(95% CI)	%	(95% CI)	%	(95% CI)	%	(95% CI)
**Proportion of physicians who would comply with the woman's request for a caesarean delivery because "this is her choice":**																
	55	(46–64)	15	(9–23)	19	(14–26)	75	(57–87)	22	(17–29)	57	(33–78)	79	(72–85)	49	(42–57)

Proportion of physicians indicating the following reasons for compliance with patient's request (*):																
Out of respect for the woman's autonomy	93	(87–97)	83	(61–94)	79	(62–90)	95	(80–99)	96	(78–99)	100		97	(92–99)	97	(93–99)
To avoid possible problems of non-compliance during delivery	45	(33–58)	40	(30–51)	53	(33–72)	49	(41–58)	37	(22–55)	62	(50–74)	33	(29–37)	52	(39–64)
To avoid possible legal consequences if something goes wrong	63	(54–71)	81	(70–89)	89	(50–99)	69	(59–77)	30	(13–53)	87	(69–96)	52	(40–63)	31	(25–37)

(*) Proportions computed on physicians who would agree to perform a caesarean delivery because it was the woman's choice. More than one reason could be quoted.

Reproduced with permission from BJOG [19]

### Value premises

Values and norms, of which ethical principles are a subclass, are crucial to the assessment of the ethical standing of the different recommendations that may ensue [23]. This analysis utilizes the principlist approach [22] to examine the ethical conflict raised by CDMR and assess what normative conclusions, if any, can be drawn. This approach was chosen for two reasons: it is probably the method most commonly taught to physicians in Europe, and is compatible with a number of ethical theories [24]. Principles are derived from common morality and may be shared by people from different times and cultures [25,26]. Thus, they are particularly appropriate when analysing differences of attitudes and practices between countries.

In line with the recommendation of the Council for International Organizations of Medical Sciences (CIOMS) we shall consider beneficence to encompass non-maleficence (do no harm) [27]. Thus, three principles will be used in this analysis: beneficence, that is minimising harm and maximising benefit; respect for autonomy, or compliance with self-determination; and justice, encompassing equity and non-discrimination.

The most important stakeholders are the pregnant woman, the foetus, and the physicians responsible for patient care. Where applicable, third party payers and society at large will be considered because of the impact on resource allocation and policy. Other health care practitioners and staff whose time, skills and resources might be used differently depending on the choices made, and the father or other relatives or carers will also be affected; however, their involvement, as far as we can see, is unlikely to raise unique challenges not considered elsewhere in this analysis.

### Principle-based ethical analysis

#### Beneficence and non-maleficence

The principle of *beneficence *(including non-maleficence) affirms the obligation on health care professionals to minimise harm and maximise benefits. Harm can only be justified if unavoidable and if it occurs during attempts to achieve greater good (mostly but perhaps not exclusively) to the individual concerned.

##### The pregnant woman

Decisions contrary to the pregnant woman's will, could negatively affect her experience of labour and her relationship with health care providers, and even with her child. Operative vaginal delivery and emergency CS, which might become necessary during labour, carry greater risk compared to elective CDMR [4,28]. But despite its increased safety, CDMR remains a non-trivial abdominal surgical procedure, which can result in short and long term harm [5,8,28,29]. It also has implications beyond the index pregnancy, such as higher morbidity and need for subsequent CS [1,10,12,30]. On the other hand, vaginal birth has been reported to result in damage to the pelvic floor and short-term urinary incontinence, but the causal link with clinically relevant symptoms is not conclusively proved [8,31,32]. Avoidance of pain during labour has also been cited as a potential maternal benefit [33], but could arguably also be addressed by due attention to pain relief in labour. Along with medical outcomes, the principle of beneficence requires consideration of any non-medical interests the woman may have. For instance, an elective CS allows better planning and avoids the uncertainty of the onset of labour.

##### The foetus

A CS is relatively safe for the foetus, but it is not without risks, particularly respiratory problems and an increased length of hospital stay [5,12,34-37]. These risks need to be weighed against those inherent in vaginal birth [38]. Breastfeeding may be adversely affected by CS [39] although data specifically related to CDMR are not available.

##### The physicians

There is lack of agreement on whether beneficence or harm to the physicians is to be considered in an ethical analysis [40]. Complications may happen in instances where CDMR is denied, and legal consequences might harm the obstetrician. In the US, a positive association between obstetricians' insurance premiums and primary caesarean delivery rates has been reported [41], and it was suggested that obstetricians may carry out non medically indicated CS to fend off liability problems [42]. In the EUROBS study, fear of legal consequences was factored in decision-making by a large number of obstetricians in all countries except the Netherlands and Sweden [19]. On the other hand, CDMR is not risk free, and despite the woman's explicit request, litigation might arise in case of adverse outcome. Finally, obstetricians' personal time management and, in some countries and settings, also financial incentives may work to favour a higher CS rate [8,43].

##### The third party payers

Judicial use of resources is probably the only relevant concern for third party payers. CS incurs higher costs compared to vaginal birth [44,45], although the difference in cost for elective caesarean section may not be substantial [46].

##### Society

At the public health level the increasing CS rates, a trend common to most countries, is a source of worry. While a variety of counter measures such as educational programs and guidelines have been proposed, CDMR has the potential to aggravate the trend, particularly as a first CS appears to be strongly predictive of subsequent cesareans. It has been argued that this could result in loss of obstetric and midwifery skills in the management of vaginal birth [47].

#### Respect for autonomy

A*utonomy *is inextricably linked to the Western tradition of liberalism, and is given central status in Kantian moral philosophy and in Mill's utilitarian liberalism [22,48]. The principle of *respect for autonomy *requires that the views of those who are capable of deliberation about their personal goals be sought and respected. As the foetus lacks such capabilities, the question of foetal autonomy does not arise.

##### The pregnant woman

There is consensus that a pregnant woman should be adequately informed about the management of delivery and the consequences of different decisions, and this pre-requisite was specified in our case scenario description. The principle of respect for autonomy could be seen to assign higher priority to a pregnant woman's wishes in case of disagreement. However, such a conclusion is compounded by conflicting views of autonomy itself, and different conclusions may be drawn depending on whether one specifies autonomy in terms of negative or positive rights: that is, the right to refuse a certain intervention, or also the right to demand it. There seems to be a generally accepted view, supported by case law, that a pregnant woman's desire for non-intervention overrides other considerations [49], but a request for intervention is more controversial [50].

##### The physicians

Good obstetric practice requires the physician to act in the best interest of both mother and foetus. To this end, a physician's autonomy and medical training require him/her to inform the patient and recommend the most appropriate course of action. Traditional medical teaching is that a surgical intervention such as CS requires justification. Thus, in case of disagreement, physician's autonomy and professional integrity allows him/her to reject the patient's request, provided that the patient is not put at risk and that timely provisions could be made for the transfer of care to another physician.

##### The third party payers

Public provisions account for the vast majority of funding for obstetric services in the countries participating in the EUROBS study. Concerns have been raised in many of these countries about the rising CS rates [51,52], but beyond general guidance to obstetricians, individual decisions are delegated to obstetricians and patients.

#### Justice

Rival theories produce different interpretations of the ethical principle of *justice*. Whilst the principle of formal justice that requires equals to be treated equally raises no disagreement, there are many different material principles of distributive justice [22]. These include – amongst others – equality, need, solidarity, contribution, merit, and even lottery. Ambivalence regarding which one should take priority underpins much of the disparities in health care provisions between, and sometimes within, countries. At least to egalitarians, justice entails an ethical obligation to equal distribution or to a fair equality of opportunity when it comes to satisfying needs. Health care needs vary between individuals, but are guided by the requirements of treatment and prevention aimed at restoring or maintaining 'typical' function (although this may not always be easily defined). Relevant to the present discussion is also justice as fair distribution of burdens and benefits, and non-discrimination. Lastly, the word "justice" is used in the context of fulfilling legal requirements (legal justice).

##### The pregnant woman

Avoiding unjust discrimination and allowing fair access to health care permit individualisation of care based on need. Many would argue that an obligation to provide such access is limited to the provision of basic need. The difficulties inherent in delimiting what constitutes basic provision invite public and policy debate and decision.

##### The foetus

It could be argued on ethical (but not always on legal) grounds, that the requirements of justice should provide the unborn with a fair opportunity at the start of life. This may not be applicable in cases of CDMR because of the absence of fetal indication for the intervention.

##### The physicians

No ethical problems need to arise here provided that the standard is uniformly applied, but the issue of legal justice is important. The different medico-legal environments in Europe may at least partly explain the differences in the obstetricians' perceptions of threat of litigation seen in the EUROBS study. The physicians may also have a role in ensuring efficient use of resources, and avoiding unnecessary expenditure.

##### Third party payers

Budgetary constraints argue against more expensive interventions unless linked to tangible benefit. Furthermore, limited resources often mean that additional expenditure in one area can have adverse impact on other services. Transparency and accountability are important means towards achieving distributive and legal justice, but are unlikely to resolve the controversies surrounding priority setting. Additional costs incurred by CDMR may impact on different groups of patients. Co-payment by patient to cover the extra costs has been suggested [6], but it would raise additional practical and ethical concerns.

## Discussion

The findings from the EUROBS study indicate that European obstetricians generally accept two propositions: woman's self-determination and medical utility. The right to self-determination is considered important, as between 79 and 97% of those who agreed to perform CS based on the woman's request quoted respect for autonomy as justification. The view that vaginal birth is preferable in the absence of medical indications for CS is evidence of the weight given to medical utility, a concept encompassing calculations of risk and benefit as well as concerns for optimising the use of resources. How are these apparently conflicting positions to be balanced?

Partly because of lack of convincing empirical evidence, the balance of our analysis of benefits and harm of CDMR does not clearly shift one way or the other. There is evidence that a spontaneous, non-operative vaginal delivery is the safest option for the mother and the neonate, but such favourable outcome cannot be predicted with certainty. Overall, the benefits and burdens of elective non labour cesarean delivery appear to equal those of planned vaginal birth, as most of the risks of caesarean section may be linked to its primary indication, or to procedures done as an emergency. However, confounding is difficult to control, and randomized comparisons of "planned vaginal delivery" versus "planned cesarean" in the absence of medical indications are not available. An additional difficulty is linked to the somewhat different types and time of occurrence of the risks. Most reviews and recently issued guidelines on the topic have therefore concluded that overall no major differences in outcome exist between CDMR and planned vaginal delivery, but the evidence is too weak to state definitely that differences are completely absent [1,10,12]. This argues against any change in the currently recommended standard of care for uncomplicated term pregnancy, i.e. vaginal delivery.

An appeal to autonomy as a basis for CDMR requires that the woman's choice is free from controlling influences [53]. Several reports have highlighted factors that may interfere with a woman's freedom of choice, including physician's convenience, medico-legal and financial interests, or a tendency to depict vaginal birth as archaic and disfiguring [43,50,54,55]. Moreover, whilst respect for autonomy entails the right to reject unwanted treatment, it does not enable a right to obtain treatment on demand.

The doctrine of informed consent requires the physician to discuss with the patient the available diagnostic and therapeutic alternatives, to explain the relevant risks and benefits, and to make recommendations [53]. Such duty would extend also to surgical delivery in the absence of medical indication, should it come to be viewed as a viable standard alternative to vaginal birth: failure to do so might be viewed as unfair to those women who would have asked for CS if aware of the option. Extravagant as it may appear, such scenario was already anticipated by a commentary in favour of CDMR: "When elective caesarean section is seen to be so safe, does not concealing this fact deprive women of an informed choice surrounding their delivery?" [56]. Such "normalization" of caesarean delivery [58], however, is not justified by the currently available evidence regarding its benefits and risks compared to vaginal birth. We therefore believe that a change in the standard of care to grant CDMR is not ethically justified.

The application of the principle-based approach has provided a useful framework for orderly analysis of the rights and duties of the parties involved taking account of available scientific evidence, and enabled us to reach a general conclusion about what should be considered an acceptable standard. On beneficence/non maleficence grounds CDMR cannot currently be considered the safest option. On autonomy grounds, concerns about the influence of interfering factors on the women's choice, and differences between positive and negative rights, argue against CDMR. Finally, on justice grounds, the view that CDMR is an acceptable alternative to vaginal birth in case of normal pregnancy would require including this information in the routine prenatal counselling, with the paradoxical consequence of supporting rather then merely accepting the practice. These arguments, based on the application of principlism analysis, have allowed to conclude that vaginal birth should remain the standard of care in uncomplicated pregnancy.

The remaining question to be answered is what to do in the cases when appropriate information and persuasion fail, and where a woman insists on cesarean delivery against the obstetrician's recommendation: a situation which is illustrated by the vignette used in the EUROBS study. Research has shown that, apart from those derived from social pressures and obstetricians' influences, spontaneous maternal requests for caesarean delivery are probably for the time being a more limited phenomenon than suggested by the amount and virulence of the debate raised around it [58]. Many cases appear to be linked to special personal circumstances, such as psychosocial difficulties, previous negative experiences, and specific fears or anxiety for vaginal birth. We believe that, in selected cases, taking into account such personal circumstances and agreeing to caesarean delivery is more beneficial than subjecting the woman to the process of vaginal birth against her will. Compliance with CDMR should however remain the exception, to be justified on the basis of special individual circumstances as those stated above. A discussion about mode of delivery should be started since early pregnancy, to allow sufficient time for listening and counselling, second opinion and even, in case of persisting disagreement, timely referral to a colleague without danger of jeopardizing the patient's care.

## Competing interests

The authors declare that they have no competing interests.

## Authors' contributions

TN conceived the paper and drafted the manuscript. MH and GL contributed to the analysis of the obstetrical literature, and RS to the interpretation of the epidemiological evidence. MDF performed the analyses of the EUROBS database. MC was principal investigator of the EUROBS project, and participated in preparing the discussion. All authors contributed to finalize the paper, read and approved the final manuscript.

## Pre-publication history

The pre-publication history for this paper can be accessed here:


